# Development and Experimental Validation of Machine Learning-Based Disulfidptosis-Related Ferroptosis Biomarkers in Inflammatory Bowel Disease

**DOI:** 10.3390/genes16050496

**Published:** 2025-04-27

**Authors:** Yongchao Liu, Jing Shao, Jie Zhang, Mengmeng Sang, Qiuyun Xu, Liming Mao

**Affiliations:** 1 Department of Immunology, School of Medicine, Nantong University, Nantong 226019, China; 2231310015@stmail.ntu.edu.cn (Y.L.); romly80@sohu.com (J.S.); zhangjie@ntu.edu.cn (J.Z.); sangmm@ntu.edu.cn (M.S.); 2 Basic Medical Research Center, School of Medicine, Nantong University, Nantong 226019, China

**Keywords:** disulfidptosis-related ferroptosis genes, inflammatory bowel disease, machine learning, biomarker

## Abstract

**Background:** Inflammatory bowel disease (IBD) is a chronic inflammatory condition of the gastrointestinal tract, defined by intestinal epithelial cell death. While ferroptosis and disulfidptosis have been linked to IBD pathogenesis, the functional significance of disulfidptosis-related ferroptosis genes (DRFGs) in this disease remains poorly characterized. This investigation sought to pinpoint DRFGs as diagnostic indicators and clarify their mechanistic contributions to IBD progression. **Methods:** Four IBD datasets (GSE65114, GSE87473, GSE102133, and GSE186582) from the GEO database were integrated to identify differentially expressed genes (DEGs) (|log2FC| > 0.585, adj. *p* < 0.05). A Pearson correlation analysis was used to link disulfidptosis and ferroptosis genes, followed by machine learning (LASSO and RF) to screen core DRFGs. The immune subtypes and single-cell sequencing (GSE217695) results were analyzed. A DSS-induced colitis *Mus musculus* (C57BL/6) model was used for validation. **Results:** Transcriptomic profiling identified 521 DEGs, with 16 defined as DRFGs. Nine hub genes showed diagnostic potential (AUC: 0.71–0.91). Functional annotation demonstrated that IBD-associated genes regulate diverse pathways, with a network analysis revealing their functional synergy. The PPI networks prioritized *DUOX2*, *NCF2*, *ACSL4*, *GPX2*, *CBS*, and *LPCAT3* as central hubs. Two immune subtypes exhibited divergent DRFG expression. Single-cell mapping revealed epithelial/immune compartment specificity. The DSS-induced murine colitis model confirmed differential expression patterns of DRFGs, with concordant results between qRT-PCR and RNA-seq, emphasizing their pivotal regulatory roles in disease progression and potential for translational application. **Conclusions:** DRFGs mediate IBD progression via multi-signal pathway regulation across intestinal cell types, demonstrating diagnostic and prognostic potential.

## 1. Introduction

Inflammatory bowel disease (IBD), encompassing Crohn’s disease and ulcerative colitis, is a progressive, immune-mediated condition affecting the intestinal tract [1,2,3]. In 2017, an estimated 6.8 million people worldwide were found to have IBD, which is a jump from the 3.7 million who were diagnosed in 1990 [4,5]. The exact cause of IBD remains unclear; however, it is believed to result from a combination of genetic susceptibility, abnormal immune system activity, and environmental triggers [6]. Several studies have indicated that a delayed diagnosis of IBD may lead to worse clinical outcomes [7]. Therefore, the early diagnosis and treatment are crucial for controlling the progression of the disease. While the current methods for early diagnosis of IBD are inadequate. Therefore, there is an urgent need for new biomarkers to enable the early identification of IBD and improve risk stratification.

Researchers have reported a novel form of cell death called “disulfidptosis” in a recent edition of *Nature Cell Biology* [8], which is a fast type of cell death brought on by disulfide stress from too much cystine accumulation during glucose deprivation, producing aberrant disulfide bonding in actin proteins and so causing actin network collapse and cell death [9]. By contrast, another controlled type of cell death brought on by iron-dependent oxidative damage is ferroptosis [10]. Ferroptosis is driven by the buildup of fatty acid peroxides resulting from dysregulated cellular iron. It is characterized by iron accumulation, GSH depletion, suppression of GPX4, and lipid peroxidation. The initiation of ferroptosis depends on the activity of several enzymes. For instance, oxidoreductases like NADPH-cytochrome P450 reductase (POR) and NADH-cytochrome b5 reductase (CYB5R1) facilitate the production of hydrogen peroxide, which, in a reaction with iron, generates hydroxyl radicals that peroxidize membrane PUFA chains, leading to ferroptosis. The knockout of these enzymes reduces hydrogen peroxide generation, thus inhibiting ferroptosis as well as the peroxidation of lipids [11]. Additionally, Wu et al. [12] demonstrated that employing AM630 (a selective CB2R inverse agonist) significantly eliminated the detrimental influence of β-caryophyllene on macrophage ferroptosis. Ferroptosis is commonly detected in the injured gastrointestinal tract of IBD patients [13]. Targeting ferroptosis has been proposed to be an effective approach for treating IBD.

A recent study by Zhang et al. [9] disclosed a partial overlap between disulfidptosis-associated genes and some ferroptosis-associated genes in hepatocellular carcinoma (HCC) and identified several disulfidptosis-related ferroptosis genes (DRFGs) as biomarkers for the diagnosis of HCC. To date, whether DRFGs impact the onset of IBD has not been investigated. In this research, we screened DRFGs using a combined dataset generated by four IBD datasets from the GEO database and explored the potential roles of these genes in multiple aspects associated with IBD development. Our study offers novel insights into mechanistic studies of IBD through investigating the roles of DRFGs and may contribute to the development of novel anti-IBD therapeutic strategies.

## 2. Materials and Methods

### 2.1. Data Collection

We obtained four expression profile datasets, including GSE65114 (control = 12, UC = 16), GSE87473 (control = 21, UC = 106), GSE102133 (controls = 12, CD = 65), and GSE186582 (control = 25, CD = 464) from the GEO database. In total, 259 ferroptosis-associated genes (FAGs) (Table S1) were sourced from the FerrDB repository (http://www.zhounan.org/ferrdb/; accessed on 1 October 2024 [14]. The disulfidptosis-associated genes (DAGs) were curated from the dataset published by Gan’s research team (Table S2) [8].

### 2.2. Data Preprocessing

Four GEO datasets (GSE65114, GEO87473, GEO102133, and GEO186582) were harmonized using ComBat algorithm via the “sva” 3.50.0 R package to mitigate batch effects and standardize cross-platform expression. Probes were annotated with standardized gene symbols, retaining only intersection genes shared across platforms. The integrated cohort comprised 651 IBD and 70 control samples. A principal component analysis (PCA) confirmed successful batch correction through pre/postprocessing dimensionality comparison.

### 2.3. Differentially Expressed Genes (DEGs)

The DEG dataset was acquired using the R package limma 3.58.1. Finally, differential analysis of the combined dataset was performed to identify differentially expressed genes (DEGs) (|log2FC| > 0.585, adj. *p* < 0.05). The DEGs were visualized using volcano and heatmap plots, generated by the ggplot 2 package.

### 2.4. Screening DRFGs in IBD

Pearson’s correlation coefficients were computed to examine potential associations between DAGs and FAGs. Subsequently, differential expression profiles of DRFGs in IBD and normal tissues were evaluated using the ‘limma’ computational framework. The correlation between DRFGs was visualized using the tidyverse and pheatmap packages.

### 2.5. Screening of Candidate Diagnostic Biomarkers by Machine Learning

The study utilized the machine learning algorithms LASSO and RF to identify diagnostic detection markers for DRFGs in IBD, enhancing data science classification and prediction capabilities. The LASSO regression method, executed via the glmnet package, was employed to select the optimal gene signature exhibiting minimal cross-validation error. The randomForest package was used to implement the RF algorithm, which was then evaluated through 5-fold cross-validation.

### 2.6. Receiver Operating Characteristic (ROC) Curves

The ‘limma’ package was employed to construct a nomogram. The pROC package was adopted to construct receiver operating characteristic (ROC) curves for DRFGs in different subgroups (Control/IBD) and assess the diagnostic performance of hub gene expression in disease diagnosis through quantification of the area under the curve (AUC).

### 2.7. Gene Set Enrichment Analysis (GSEA)

The R package “org.Hs.eg.db” was utilized in the Gene Set Enrichment Analysis (GSEA) to investigate biological behaviors and signaling pathways [15]. The c5.go.Hs.symbols and c2.cp.kegg.Hs.symbols gene sets were downloaded from the Molecular Signatures Database (MSigDB) (https://www.gsea-msigdb.org/gsea/msigdb/; accessed on 1 November 2024).

### 2.8. Gene Set Variation Analysis (GSVA)

The GSVA algorithm represents a nonparametric, unsupervised computational approach designed to quantify the pathway enrichment levels of predefined gene collections within transcriptomic profiling datasets [16]. The GSVA method transforms gene-centric variations into pathway-centric alterations to determine the functional activity of specimens. For this investigation, curated gene collections were retrieved from the molecular signature repository, and the GSVA computational framework was employed to quantify the enrichment levels of each collection, thereby examining the underlying functional pathway variations among heterogeneous specimen groups.

### 2.9. GO and KEGG Analysis

The cluster Profiler R package was used for functional enrichment analysis of DEGs to assess the biological functions of genes and their associated pathways [17].

### 2.10. Correlation Assessment of Genes

Leverage the pheatmap package to generate a heatmap illustrating gene correlations, harness the ggpubr and limma packages for visualizing gene interplay, and adopt the circlize package to construct a chord diagram representing interconnections. Protein-protein interaction (PPI) networks linked to DRFGs were generated via the STRING database, with edges filtered by a high-confidence threshold (interaction score > 0.4).

### 2.11. Immune Cell Infiltration (ICI) Assessment and Identification of Co-Characteristic Genes

The investigation quantified immune cell subtype distributions in biospecimens using the CIBERSORT algorithm, contrasted immune cell infiltration (ICI) disparities between pathological and control cohorts, and interrogated their association with molecular co-drivers. Spearman’s rank-order analysis was applied to delineate connectivity dynamics between immunocyte populations and nodal genetic regulators, computationally modeling cross-modulation patterns across these biological units.

### 2.12. Unsupervised Cluster Analysis

The investigation employed unsupervised hierarchical clustering methodologies combined with PCA to detect significantly upregulated DRG-FRGs in 721 IBD samples (after removing the control group) by the R package ConsensusClusterPlus 1.66.0 [18], enriched molecular pathways, and differences in immune cell infiltration.

### 2.13. Single-Cell RNA Sequencing Analysis

In this investigation, we conducted rigorous data curation and downstream analysis of 43,023 single cells using Seurat R package (v5.1.0). Suboptimal cellular profiles were systematically excluded through application of predefined exclusion thresholds: cells with 300 or 3000 detected genes (to exclude low-grade cells and potential cell doublets); cells with >10% mitochondrial gene expression (indicating cellular stress/apoptosis); cells with >5% erythrocyte markers (to remove red blood cells lacking nuclear content). After filtering, 42,503 high-confidence cells were retained for further analysis. The data were log-normalized (LogNormalize), and the top 2000 highly variable genes (HVGs) were selected for PCA-based dimensionality reduction. Batch effects were corrected using CCA integration, and the top 20 principal components (PCs) were used for UMAP clustering at a resolution of 0.2, resulting in 13 distinct cell subpopulations. To annotate cell types, cluster-specific marker genes were identified using FindAllMarkers (minimum expression fraction ≥ 25%, logFC ≥ 0.25). The top 10 marker genes from each cluster were cross-referenced with the CellMarker 2.0 database for cell type identification (Table S3).

### 2.14. Experimental Animals and DSS-Induced Colitis

Male *Mus musculus* (C57BL/6) mice aged eight weeks (specific pathogen-free grade) were procured from GemPharmatech, Co., Ltd. (Nanjing, China). All rodents were housed under sterile containment conditions with strictly regulated environmental parameters: ambient temperature maintained at 22 ± 2 °C, humidity controlled at 50% ± 10%, and diurnal illumination cycles (12 h light/12 h darkness). Throughout the investigative timeline, unrestricted availability of standard chow and purified water was ensured for all experimental subjects.

Male C57BL/6 mice were randomly allocated to two cohorts s (*n* = 4 per cohort): a reference cohort and a DSS intervention cohort. The reference cohort received ad libitum provision of bi-distilled aqueous solution, whereas the DSS cohort was administered 3% DSS (prepared in bi-distilled water) throughout a 7-day exposure period (from day 0 through day 7). To assess the progression of colitis, daily evaluations of diarrhea, rectal bleeding, and body weight changes were conducted. The disease activity index (DAI) was derived following an established protocol [19]. Finally, mice were euthanized in a carbon dioxide chamber. The colons were carefully removed and rinsed with PBS. A segment was preserved in 4% paraformaldehyde for histomorphological examination, whereas the remaining tissue was cryopreserved at −80 °C for biochemical assays. All experimental protocols and animal welfare practices were sanctioned by the Institutional Animal Care and Use Committee of Nantong University (Approval No. S20211204-001).

### 2.15. Quantitative Polymerase Chain Reaction (qPCR)

Total cellular RNA was extracted from mouse colon tissues using the RNeasy Mini Kit. Complementary DNA was synthesized with HiScript III RT SuperMix for qPCR (+gDNA wiper) (R323-01, Vazyme, Nanjing, China), followed by amplification using ChamQ Broad-Spectrum SYBR Green qPCR Reaction System (Q711-02, Vazyme, Nanjing, China). Sequence-specific oligonucleotides employed in qRT-PCR assays are systematically cataloged in Table 1. Results were calculated using relative quantitative analysis of 2^−∆∆CT^, and all experiments were conducted in triplicate.

### 2.16. Statistical Analysis

Data processing, statistical analysis, and plotting in this study are performed using R 4.3.2 software. Statistical significance was defined as *p* < 0.05.

## 3. Results

### 3.1. Technology Roadmap

**Figure 1:**
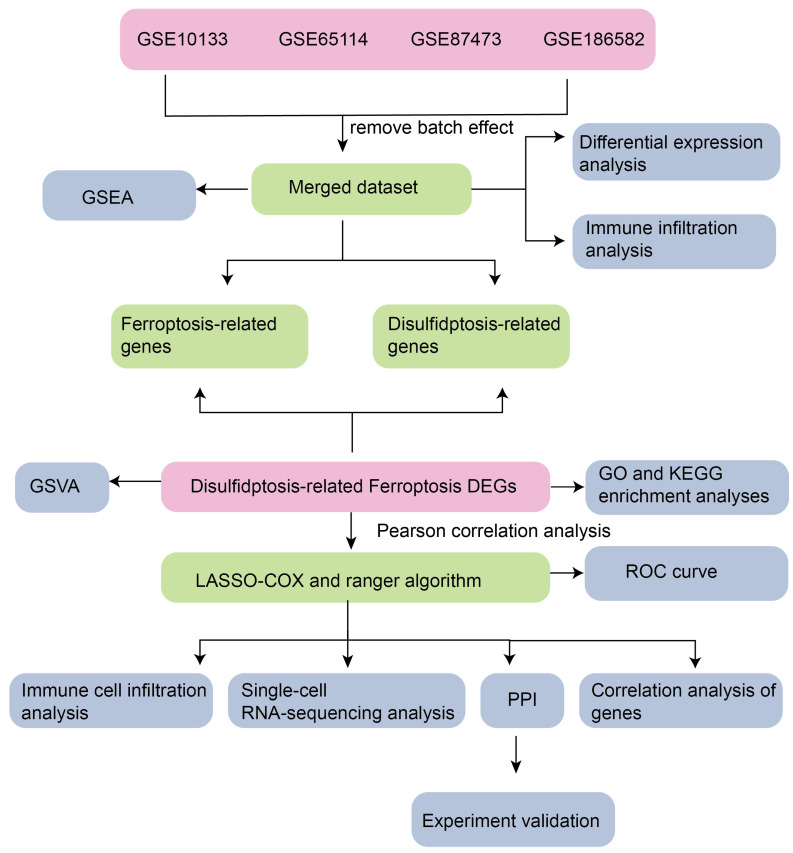
Technology roadmap. GSEA: gene set enrichment Analysis. GSVA: gene set variation Analysis DEGs: differentially expressed genes. ROC: receiver operating characteristic. PPI: protein–protein interaction.

The experimental schema of the current investigation is depicted in Figure 1.

### 3.2. Batch Removal for Dataset Integration

**Figure 2:**
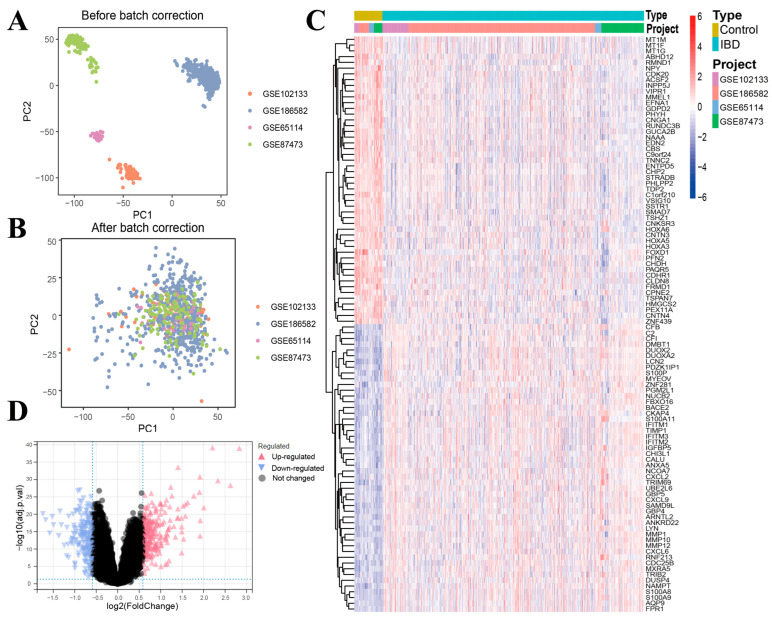
Characterization of transcriptional alterations in IBD cohorts. (**A**) Principal component projection of pre-normalization aggregated data collection. (**B**) Post-correction principal component spatial distribution of the consolidated data cohort. (**C**) Thermographic matrix and differential expression scatter plot (**D**) illustrating stratified transcriptional signatures between IBD and reference cohorts within the integrated data matrix.

Initially, we employed the R package sva to adjust for batch effects in the microarray GSE65114, GSE87473, GSE102133, and GSE186582 datasets, creating a combined GEO dataset. Following batch effect correction, we assessed variations between pre- and post-normalization datasets via PCA visualizations (Figure 2A,B). A total of 521 DEGs were identified from the metadata using the screening criteria of an adjusted *p*-value < 0.05 and an absolute log2FC > 0.585. Among these DEGs, 309 genes displayed significant upregulation in the IBD samples, while 212 genes exhibited significant downregulation, as illustrated in Figure 2C,D.

### 3.3. Screening of DRFGs in IBD

A bivariate correlation framework (Pearson’s method) was applied to examine the associations between the DRGs and FRGs. Our analysis revealed 371 molecular candidates (Table S4) demonstrating statistically significant associations (*p* < 0.05), with absolute correlation coefficients exceeding 0.3. A subsequent computational interrogation using the “limma” bioinformatics toolkit evaluated the transcriptional profiles of these DRFGs across IBD lesions and non-pathological tissues.

**Figure 3:**
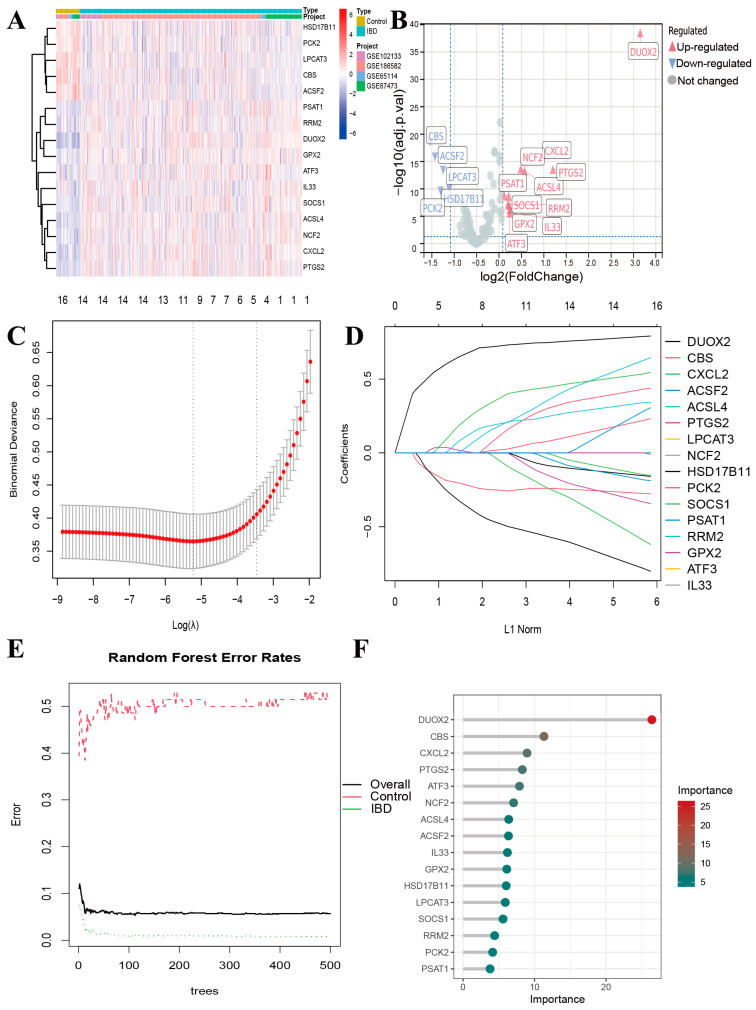
Identification of key DRFGs by machine learning. (**A**) Hierarchical clustering heatmap and dispersion plot (**B**) illustrating DRFGs between IBD and control cohorts in the integrated dataset. (**C**) Optimization of the tuning parameter λ in the Lasso regression framework via k-fold cross-validation. (**D**) Trajectory analysis of variable coefficients. (**E**) Iterative model training protocol for RF. (**F**) Feature selection methodology using the RF algorithm.

We found 16 differentially expressed DRFGs in the IBD samples compared with the normal samples (Figure 3A,B). This study utilized two machine learning algorithms, LASSO-COX regression and Random Forest, to identify the core DRFGs. Employing a LASSO-Cox regression model, we extracted 9 prognostic genetic markers from an initial pool of 16 candidates (Figure 3C,D). In contrast, the Random Forest analysis revealed 16 genes (importance scores > 2.0) as potential diagnostic biomarkers for IBD (Figure 3E,F).

### 3.4. Gene Expression of DRFGs

**Figure 4:**
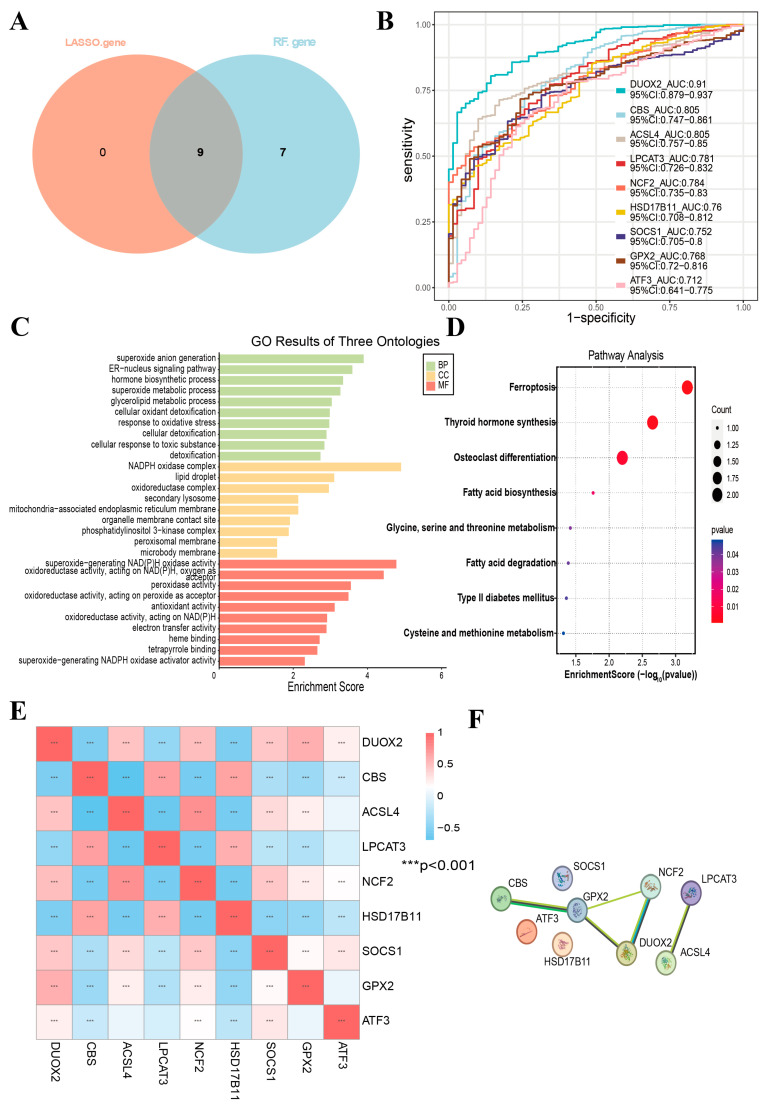
The differential expression levels of DRFGs in IBD patients. (**A**) Venn diagram showing the intersection of characteristic genes obtained by the 2 indicated algorithms in IBD patients. (**B**) ROC validation of *DUOX2*, *ACSL4*, *ATF3, NCF2*, *SOCS1*, *GPX2*, *CBS*, *LPCAT3*, and *HSD17B11* in the combined dataset. (**C**) Functional annotation clustering of DRFGs via GO term enrichment. (**D**) Pathway topology analysis of DRFGs using KEGG pathway mapping. (**E**) Correlation heat map of DRFGs. (**F**) PPI network for DRFGs.

The DEGs with signature genetic elements selected by the LASSO regression and RF methodologies revealed nine core genes (*DUOX2*, *ACSL4*, *NCF2*, *SOCS1*, *GPX2*, *ATF3*, *CBS*, *LPCAT3*, and *HSD17B11*) associated with the DRFGs (Figure 4A). To investigate the diagnostic effectiveness of the genes (*DUOX2*, *ACSL4*, *NCF2*, *SOCS1*, *GPX2*, *ATF3*, *CBS*, *LPCAT3*, and *HSD17B11*), an ROC analysis was performed on a combined dataset (Figure 4B). The results demonstrated that the following genes exhibited moderate diagnostic accuracy in distinguishing between the IBD and normal groups: *DUOX2* demonstrated diagnostic efficacy (AUC = 0.910), *CBS* exhibited a discriminatory capacity of 0.805, *ACSL4* showed a classification performance of 0.805, *LPCAT3* displayed a predictive utility of 0.781, *NCF2* achieved a receiver operating characteristic value of 0.784, *HSD17B11* attained an area under the curve metric of 0.76, *SOCS1* showed an ROC-AUC score of 0.752, *GPX2* yielded a biomarker accuracy of 0.768, and *ATF3* registered a diagnostic potential of 0.712.

### 3.5. Gene Ontology (GO)-Based Functional Annotation Profiling and KEGG Pathway Ontology Mapping

In addition, functional annotation profiling and pathway ontology mapping were conducted on the DRFGs. The GO analysis encompasses three domains (Figure 4C): Biological Process (BP), Cellular Component (CC), and Molecular Function (MF). The BP analysis revealed significant enrichment in the positive modulation of superoxide anion generation, and the ER-nucleus signaling pathway was significantly enriched. In the CC analysis, the NADPH oxidase complex and lipid droplets ranked among the top enriched terms. Furthermore, superoxide-generating NAD(P)H oxidase activity and oxidoreductase activity, acting on NAD(P)H, with oxygen as acceptor, played an essential role in MF. The outcomes of the KEGG pathway characterization demonstrated that DRFGs predominantly cluster within the ferroptosis, thyroid hormone biosynthesis, and osteoclast maturation pathways (Figure 4D).

### 3.6. Correlation Analysis and the Functional Similarity Analysis of DRFGs

To investigate the significance and functional roles of DRFGs (*DUOX2*, *ACSL4*, *NCF2*, *SOCS1*, *GPX2*, *ATF3*, *CBS*, *LPCAT3*, and *HSD17B11*) in the combined dataset, we generated correlation heat maps (Figure 4E), revealing potential interactions among these genes that may contribute to IBD progression. Additionally, the PPI analysis using the STRING database identified interactions among the nine DRFGs, leading to the construction of a PPI network (Figure 4F).

### 3.7. Enrichment Analysis of Functions and Pathways

The functional roles and molecular signaling cascades linked to signature genes were evaluated through a GSEA. The expression of genes in the control group significantly correlated with the alcohol metabolic process, cellular lipid metabolic process, a cluster of actin-based cell projections, and mitochondrial matrix (Supplementary Figure S1A). The expression of genes in the IBD group significantly correlated with the antimicrobial humoral response, humoral immune response, inflammatory response, and response to chemokines, and extracellular matrix structural constituent (Supplementary Figure S1B).

The transcriptomic profiles of the control group exhibited significant associations with the tricarboxylic acid (TCA) cycle; cytochrome P450-mediated xenobiotic metabolism; glycine, serine, and threonine biosynthetic pathways; inositol phosphate metabolic processes; and phosphatidylinositol signaling cascades (Supplementary Figure S1C). In contrast, the IBD cohort demonstrated robust correlations with cell adhesion molecule dynamics, chemokine-mediated signaling networks, complement and coagulation pathways, cytokine–cytokine receptor interactions, and the toll-like receptor signaling pathway (Supplementary Figure S1D).

### 3.8. Functional Pathway Enrichment Analysis in the Merged Dataset

We systematically investigated the pathway associations of the nine hub genes in IBD and their mechanistic roles in disease progression. The GSVA revealed distinct pathway activation profiles: *DUOX2* upregulation (Supplementary Figure S2A) predominantly correlated with cytochrome P450-mediated xenobiotic metabolism, aminoacyl-tRNA biosynthesis, and glycine–serine–threonine biosynthetic pathways. *ACSL4* overexpression (Supplementary Figure S2B) exhibited strong linkage to phosphatidylinositol-mediated signal transduction, inositol phosphate homeostasis, and synaptic plasticity mechanisms. *NCF2* elevation (Supplementary Figure S2C) demonstrated enrichment in synaptic potentiation cascades, inositol phosphate regulatory networks, and phosphatidylinositol signaling cascades. *SOCS1* amplification (Supplementary Figure S2D) was primarily associated with branched-chain amino acid catabolism, short-chain fatty acid metabolism, and xenobiotic detoxification pathways. *GPX2* upregulation (Supplementary Figure S2E) significantly modulated autophagic flux regulation, calcium-dependent signaling, and intercellular communication pathways. *ATF3* overexpression (Supplementary Figure S2F) showed predominant involvement in meiotic regulation, DNA mismatch repair, and branched-chain amino acid degradation. *CBS* downregulation (Supplementary Figure S2G) was markedly linked to histidine metabolic processes, synaptic plasticity mechanisms, and selenocysteine biosynthesis. *LPCAT3* suppression (Supplementary Figure S2H) revealed associations with TCA cycle dynamics, retinoid metabolic pathways, and glycine–serine–threonine homeostasis. *HSD17B11* reduction (Supplementary Figure S2I) displayed connections to amino acid metabolic networks, vitamin A metabolism, and peroxisomal functional pathways.

### 3.9. Analysis of Immune Cell Infiltration Patterns in IBD

**Figure 5:**
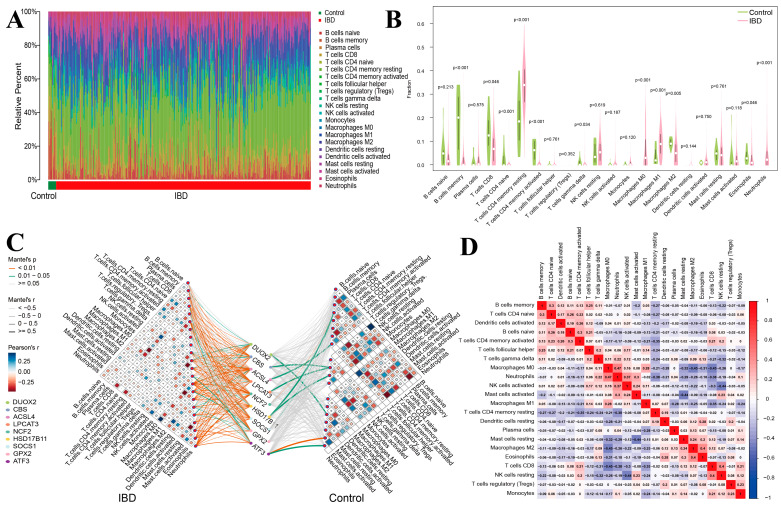
Assessment of immune cell distribution profiles in IBD. (**A**) Bar graphs of the accumulation of 22 immune cells in different samples of the dataset, with different-colored long bars representing different immune cells. (**B**) Differences in the abundance of 22 immune cell enrichment between the control and IBD groups. (**C**) Differential immune cell infiltration patterns linked to DRFGs in the non-diseased cohort relative to the IBD cohort. (**D**) The interactions among the 22 immune cell types in control group compared to IBD group.

Employing the CIBERSORT computational framework, we quantified the infiltration proportions of 22 distinct immune cell subtypes across diverse biospecimens. These immune cell abundance profiles were visualized through a composite bar chart (Figure 5A) and distribution density plot (Figure 5B), revealing notable disparities in immunocyte infiltration between healthy controls and IBD cohorts. Specifically, the IBD group exhibited elevated infiltration of resting CD4^+^ memory T cells, M0/M1 polarized macrophages, and neutrophils, whereas reduced abundance was observed for CD8^+^ T cells, memory B cells, naïve CD4^+^ T cells, activated CD4^+^ memory T cells, γδ T cells, and eosinophils. Intercellular network analyses delineating immunocyte interplay within healthy controls and IBD cohorts are illustrated in Figure 5C.

The onset of inflammatory processes can trigger ICI assessment, whereas immunocyte activation may reciprocally modulate inflammatory cascade dynamics [20]. To elucidate the interplay between expression profiles of DRFGs and immune cell subpopulations in healthy controls versus IBD cohorts, a bivariate correlation analysis was performed (Figure 5D).

### 3.10. Immune Subtypes and Hub Gene Correlation

**Figure 6:**
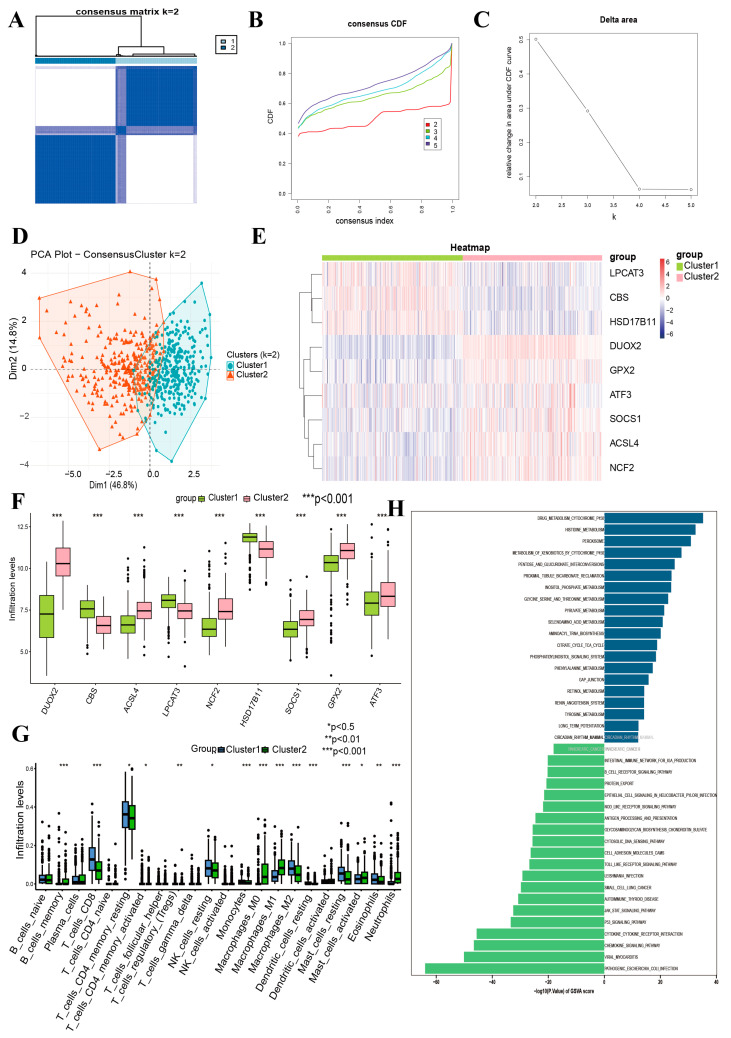
Identification of DRFGs in IBD, and immune cell infiltration and GSVA among two subtypes. (**A**) Consensus heatmaps when k  =  2. (**B**) Cumulative distribution function (CDF) plot. (**C**) Delta plot of the area under the CDF curve. (**D**) PCA of 2 subtypes. (**E**) Heatmap visualization illustrates differential transcriptomic profiles of 9 DRFGs across subtypes. (**F**) Comparative expression plots depict quantitative variations in 9 DRFGs between the two molecular subgroups. (**G**) Box-and-whisker diagrams highlight subtype-specific disparities in immune cell infiltration patterns. (**H**) Pathway enrichment comparison via GSVA contrasts cluster 1 and cluster 2.

Leveraging the expression profiles of nine DRFGs, we executed consensus clustering-based stratification of 651 IBD samples (excluding controls). The immunophenotypic classification of immune cell infiltration matrices via unsupervised consensus clustering delineated two molecular subtypes (Figure 6A): cluster 1 (327 IBD specimens) and cluster 2 (324 IBD specimens). Figure 6B,C depict the cumulative distribution function (CDF) graph and the difference plot of the integral beneath the CPF curve across multiple cluster sizes in the consensus clustering outcomes. Subsequently, the PCA (Figure 6D) revealed distinct differences among these three clusters. Comparative boxplot visualizations and expression heatmap representations (Figure 6E,F) demonstrated distinct transcriptional profiles, with a notable upregulation of *DUOX2*, *ACSL4*, *NCF2*, *SOCS1*, *ATF3*, and *GPX2* in cluster 2, alongside a significant downregulation of *CBS*, *HSD17B11*, and *LPCAT3* within the same cluster.

The CIBERSORT computational framework was employed to perform immune cell profiling of clusters C1 and C2. As demonstrated in Figure 6G, the immunocyte infiltration abundance exhibited marked disparities between the two subtypes, particularly in terms of memory B cells, CD8^+^ T cell subsets (resting/activated memory phenotypes), T Regulatory cells (Tregs), resting NK cells, mononuclear phagocytes (M0/M1/M2 polarized macrophages), mast cell activation states (resting vs. activated), and granulocyte populations (eosinophils and neutrophils). Finally, the GSVA (Figure 6H) mainly revealed that cluster 2 was more enriched in DRUG_METABOLISM_CYTOCHROME_P450, HISTIDINE_METABOLISM, and PEROXISOM compared to cluster 1.

### 3.11. Spatial Mapping Profiling via Single-Cell Transcriptomic Datasets

**Figure 7:**
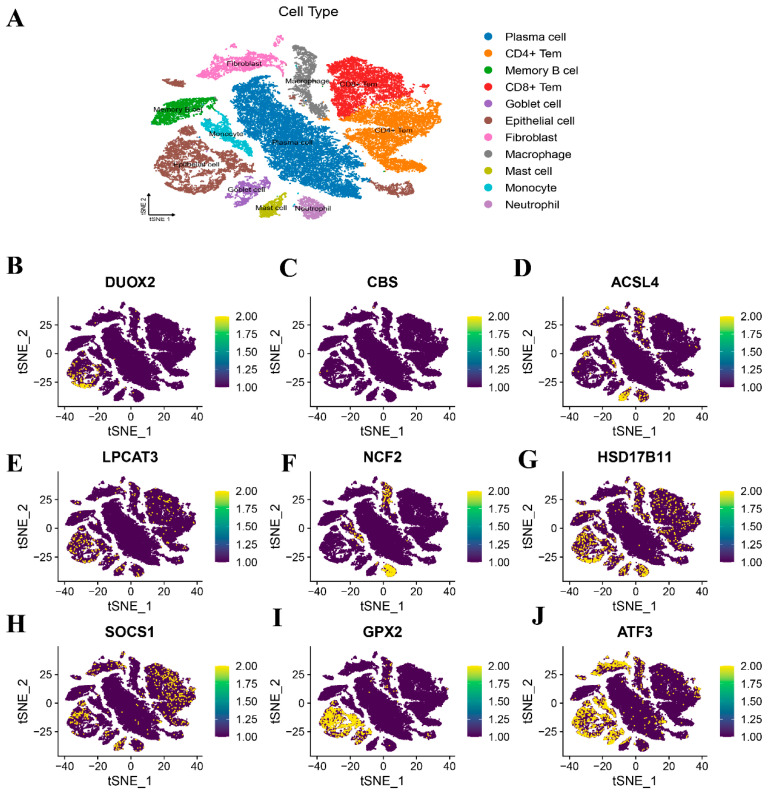
Single-cell transcriptomic datasets were analyzed to delineate cellular architecture. (**A**) t-SNE projection with lineage-specific cluster labeling. (**B**–**J**) Transcript abundance of nine DRFGs across phenotypically defined cellular subsets.

To delineate the cellular specificity of the nine DRFGs, we performed single-cell transcriptomic profiling across diverse cell populations. The IBD single-cell dataset GSE217695 (18 samples) was interrogated, revealing minimal batch effects (Supplementary Figure S3A), thus enabling robust downstream characterization. Cellular taxonomy resolution via unsupervised clustering partitioned all of the cells into 12 transcriptional clusters, ultimately annotating 11 distinct cell lineages through surface marker-guided classification (Figure 7A; Supplementary Figure S3B). The spatial expression mapping revealed cell-type-restricted enrichment patterns: *DUOX2*, *GPX2*, and *LPCAT3* were predominantly localized to memory B cells (Figure 7B,I,E), *CBS* were found on CD8^+^ Tems (Figure 7C), *ACSL4* appeared on fibroblasts (Figure 7D), *NCF2* and *HSD17B11* were mainly expressed on monocytes (Figure 7F,G), and *ATF3* demonstrated goblet cell predominance (Figure 7J).

### 3.12. Validation of DRFGs in Mouse Colonic Tissues of DSS-Mediated Colitis

**Figure 8:**
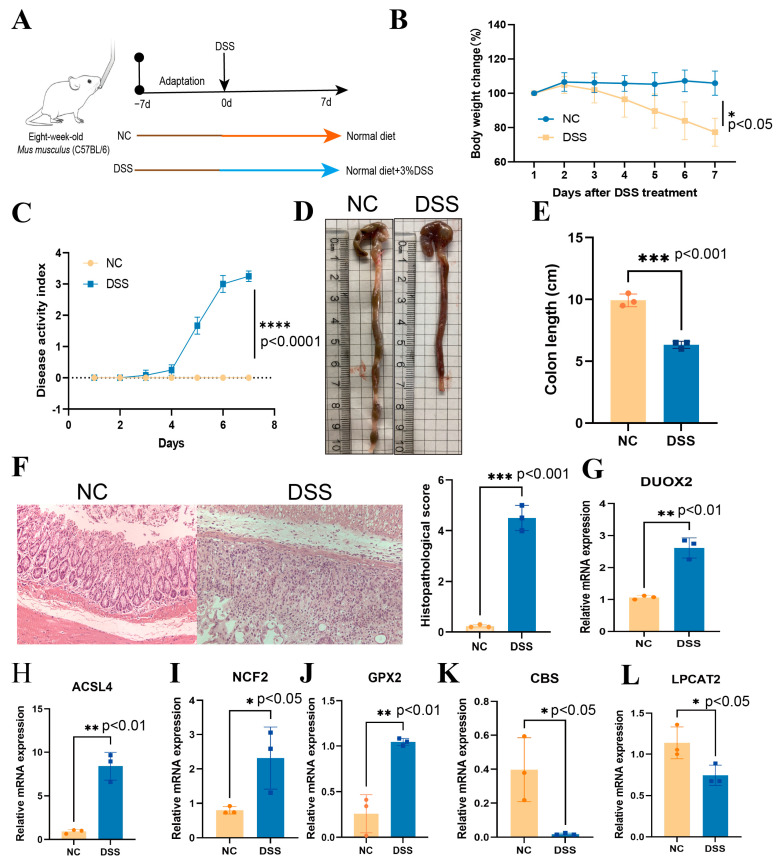
Transcriptional profiles of select DRFGs were experimentally confirmed in DSS-mediated murine colonic tissues. Pathological hallmarks of DSS-triggered colitis are shown in the following: (**A**) schematic workflow of experimental colitis induction; (**B**) body weight dynamics (% baseline); (**C**) DAI progression; (**D**,**E**) colon shortening quantification; (**F**) hematoxylin–eosin histopathology (200× magnification) with gross pathology assessment scores. qRT-PCR validation revealed differential transcript levels: *DUOX2* (**G**), *ACSL4* (**H**), *NCF2* (**I**), and *GPX2* (**J**) exhibited marked upregulation (*p* < 0.05), while *CBS* (**K**) and *LPCAT3* (**L**) showed significant downregulation in DSS-treated versus control cohorts. Values represent mean ± SD (*n* = 8 biological replicates). (* *p* < 0.05, ** *p* < 0.01, *** *p* < 0.001, **** *p* < 0.0001).

To experimentally corroborate computational predictions, the C57BL/6 mice were administered DSS to generate a colitis model (Figure 8A). The DSS-exposed cohorts exhibited significant weight loss, elevated disease activity indices (DAIs), colon shortening, and exacerbated histopathological scores (Figure 8B–F), confirming the model validity. The colonic tissues of mice were collected and the mRNA expression levels of the DRFGs involved in PPI were examined using qRT-PCR. *DUOX2*, *ACSL4*, *NCF2*, and *GPX2* exhibited extreme upregulation (log2FC > 585, adj. *p* < 0.05) across both RNA-seq predictions and qRT-PCR validations. Their cross-platform consistency underscores their reliability as core regulators in colitis (Figure 8G–L, Table S5). *CBS* and *LPCAT3* showed profound suppression (log2FC < −585, adj. *p* < 0.05). These results underscore the central functions of these DRFGs in colitis pathogenesis and strengthen their candidacy as reliable disease-associated markers or intervention targets. Specifically, the exceptionally high log2FC values (|log2FC| > 585) observed across both the computational and experimental platforms solidify their diagnostic utility and pathomechanistic relevance, positioning them as prime candidates for further translational exploration.

## 4. Discussion

Despite notable progress in anti-IBD therapeutics and optimized therapeutic protocols, the worldwide incidence of IBD has surged significantly, particularly in industrialized nations and developing regions [21]. This epidemiological shift is strongly linked to industrialization-associated environmental triggers [21]. The pathogenesis of IBD involves complex interactions among multiple molecular pathways, significantly impacting disease relapse and complications in patients [22]. In IBD, intestinal epithelial cell death occurs through intricate and interdependent mechanisms, with extensive crosstalk between cells under pathological conditions [23,24]. Exploring diverse modes of programmed cell death may provide a novel therapeutic avenue for IBD management. Herein, we evaluated the functional significance of a gene cluster implicated in modulating two distinct regulated cell death pathways: disulfidptosis and ferroptosis. These genes are referred to as DRFGs. Both disulfidptosis and ferroptossis are closely associated with cellular redox homeostasis and have been implicated in the pathogenesis of IBD [8,25,26]. Investigating the roles of these genes in IBD may uncover novel regulatory mechanisms and identify potential therapeutic targets for the pathogenesis of the disease. Therefore, through analyzing four datasets of IBD patients from the GEO database, we identified several differentially expressed DRFGs in affected individuals relative to healthy controls. Further analyses showed that these genes interplay closely and are enriched in multiple biological processes and play roles in various signaling pathways. The PPI profiling revealed significant connectivity among *GPX2*, *DUOX2*, *ACSL4*, *NCF2*, *LPCAT3*, and *CBS*, with these regulators forming a cohesive functional cluster. The single-cell sequencing data showed that the differentially expressed DRFGs exhibited distinct expression patterns in colonic tissue cells. The experimental validation using a DSS-triggered murine colitis model demonstrated concordance between the DRFG transcriptional profiles and computational predictions.

Disulfidptosis is an emerging cellular death mechanism characterized by the abnormal formation and accumulation of intracellular disulfide bonds, which are induced by oxidative stress due to an imbalance in redox homeostasis [8]. The buildup of proteins with disulfide bonds can lead to disulfide stress and cell death. In comparison, ferroptosis represents an iron-catalyzed form of cell death instigated by lipid peroxide overload. The initial step of ferroptosis is the malfunction of the glutathione-dependent antioxidant system [27]. Both modalities of cellular demise converge on perturbing the homeostatic equilibrium of redox regulation. As a specific oxidative insult, disulfide stress-mediated disulfidptosis induces antioxidant defense collapse and ROS accrual, thereby exacerbating oxidative burden and synergizing ferroptotic execution. Consequently, the genetic determinants modulating disulfidptosis constitute a unique subset of ferroptosis-associated genes. Investigating the roles of these genes in the progression of ferroptosis may deepen our understanding of the crosstalk between disulfidptosis and ferroptosis. Accumulating evidence has demonstrated that glucose metabolism and glucose consumption are markedly increased within the colonic epithelial lining of IBD cohorts [28]. The epithelial damage and persistent diarrhea may lead to reduced glucose absorption in the intestine. Therefore, the intestinal mucosa of IBD patients may be in a glucose-lacking state. Furthermore, the expression levels of the glutamate–cystine antiporter SLC7A11 and the cystine abundance in the intestinal mucosa of IBD patients exhibit pronounced upregulation in ulcerative colitis (UC) tissue relative to healthy controls [29]. This suggests that excessive cystine in the UC mucosa may drive disulfide accumulation, precipitating disulfide stress and subsequent disulfidptosis in the intestinal lining. As discussed above, the occurrence of disulfide stress in the intestinal mucosa could enhance oxidative stress in the cells and promote the initiation of ferroptosis. Consequently, genes implicated in disulfidptosis modulation may influence IBD pathogenesis by orchestrating the induction of both disulfidptosis and ferroptosis. Nevertheless, the precise functions and molecular pathways mediated by DRFG sets in IBD remain elusive, necessitating in-depth mechanistic exploration.

In this study, we integrated GSE65114, GSE87473, GSE102133, and GSE186582 to obtain a combined dataset. The GSEA results in the IBD group indicate significant enrichment of antimicrobial humoral response, humoral immune response, inflammatory response, response to chemokines, and extracellular matrix structural constituent genes in the combined dataset. Additionally, employing Spearman’s rank correlation, transcriptomic profiling, the Random Forest algorithm, and univariable survival analysis, we identified nine DEGs, which could be prognostic markers in disulfidptosis-related ferroptosis. The KEGG functional annotation revealed a significant enrichment of these genes in ferroptosis-related pathways. The GO enrichment analysis revealed that these nine DRFGs exhibit enriched functions associated with NADPH oxidase complex and superoxide-generating NAD(P)H oxidase activity. Some argue that NADPH is crucial in regulating ferroptosis by supplying reducing equivalents for antioxidant defenses and facilitating ROS production, and its levels can serve as biomarkers for predicting cellular sensitivity to ferroptosis [30]. Six hub genes were screened and the validation of the datasets revealed that *DUOX2*, *ACSL4*, *NCF2*, *GPX2*, *CBS*, and *LPCAT3* had positive diagnostic effects on IBD.

Almost all the hub genes, in line with our research, have shown an association with IBD. DUOX2 is a key member of the NADPH oxidase family [31]. *DUOX2* in intestinal epithelial cells regulates host–microbiome interactions and homeostasis; its deletion alters the microbiome and reduces colitis susceptibility by limiting ROS-mediated damage, suggesting DUOX2 as a therapeutic target for intestinal inflammation [32]. *DUOX2* may influence IBD through drug metabolism; cytochrome p450; aminoacyl tRNA biosynthesis; and glycine, serine, and threonine metabolism. *ACSL4* regulates cellular sensitivity to ferroptosis by modulating the composition of cell membrane lipids, especially the levels of polyunsaturated fatty acids [33]. Within a murine colonic inflammation paradigm established via 3% DSS exposure, the *ACSL4* expression increases. The administration of Ferrostatin-1 mitigated DSS-triggered colonic inflammation and suppressed ACSL4 protein levels [34]. ACSL4 may influence IBD through the phosphatidylinositol signaling system, inositol phosphate metabolism, and long-term potentiation pathways. *NCF2* is a gene that encodes the p67phox protein, which is a subunit of the NADPH oxidase complex [35]. *NCF2* is associated with susceptibility to IBD, with studies indicating that mutations in *NCF2* may alter NADPH oxidase activity and ROS production, potentially causing immune system overreaction or dysfunction, leading to chronic intestinal inflammation in IBD [36,37,38]. The excessive production of ROS can lead to lipid peroxidation of cell membranes, thereby inducing ferroptosis. *GPX2* exhibits consistent upregulation under conditions of oxidative homeostasis disruption [39]. GPX2 is selectively enriched within the gastrointestinal mucosal lining, with augmented expression observed in pathologies including IBD and colorectal malignancies [40]. High expression of GPX2 in lung cancer promotes ferroptosis [41]. In UC patients, inflammation-induced intestinal barrier damage is exacerbated by reduced *CBS* expression and hypermethylation of the CBS promoter within inflamed colonic mucosa relative to adjacent non-inflamed regions [42]. Moreover, Zheng et al.’s investigation revealed that apolipoprotein C1 (*APOC1*) induces resistance to ferroptosis by increasing the expression of CBS [43]. *LPCAT3* modulates ferroptotic sensitivity by altering the phospholipid composition in cellular membranes [44]. Alterations in LPCAT3 enzymatic activity can perturb polyunsaturated fatty acid (PUFA) incorporation into phospholipids, rendering them susceptible to peroxidative damage [45]. Enhanced lipid peroxidation precipitates membrane destabilization and cell death, thereby exacerbating the inflammatory sequelae and tissue injury characteristic of IBD.

The ICI analysis revealed that the IBD cohort demonstrated a statistically significant elevation in resting memory CD4^+^ T cells, M0 macrophages, M1 macrophages, and neutrophils, concurrent with a marked reduction in CD8^+^ T cells, memory B cells, naïve CD4^+^ T cells, activated memory CD4^+^ T cells, γδ T cells, and eosinophils. The alterations of some of the infiltrating cells observed in our analysis were in agreement with some previous studies. For instance, in inflamed intestines of IBD patients, macrophages predominantly exhibit the M1 phenotype, secreting pro-inflammatory cytokines that promote disease development, accompanied by a reduction in M2 macrophages [46,47]. Alba Garrido-Trigo and colleagues found that, besides increased macrophage heterogeneity in IBD, a subset of human angiogenic neutrophils was observed at inflammatory sites [48]. It was found that CD4 and occasional CD8 memory T cell responses to intestinal bacteria are present in healthy blood, while memory B cell responses are limited in IBD [49]. IBD patients show increased *IL-15Rα* mRNA in mucosal B cells, indicating *IL-15*′s role in inflammation [50]. Hence, the present results align with prior investigations and highlight the critical role of these cellular subsets in IBD pathogenesis, as evidenced by computational biology approaches.

This investigation has inherent methodological constraints. Initially, supplementary experiments are essential to validate the functional significance of the core genes in ferroptosis regulation and IBD pathogenesis. Although gene expression patterns have been verified through PCR analysis in colonic tissues from DSS-challenged murine colitis models, expanded in vitro and in vivo studies are warranted to elucidate the molecular pathways governed by these key regulators. Moreover, the immunological alterations associated with ferroptosis in IBD necessitate rigorous experimental corroboration.

In conclusion, our study identified 16 differentially expressed DRFGs in IBD patients. Using two machine learning algorithms, we identified nine core DRFGs. These hub genes exhibit moderate diagnostic accuracy in distinguishing between IBD and normal groups. The signature genes in IBD are enriched in multiple biological processes and play roles in various signaling pathways. Based on the expression of nine DRFGs, two immune signature subgroups were identified. Single-cell sequencing analysis of the IBD dataset revealed differentially expressed DRFGs with distinct expression patterns in colonic tissue cells. The expression patterns of six DRFGs were validated in mouse colonic tissues of a DSS-mediated model. The DRFGs identified in our study may influence IBD pathogenesis through the modulation of diverse signaling cascades across heterogeneous intestinal cellular subsets, thereby serving as potential prognostic biomarkers for IBD.

## Figures and Tables

**Table 1:** The primers used in qPCR analysis.

Genes	Sequences
*DUOX2*	F: CCT GCT CTC CTT GGT CCC TGT C
	R: AGT TCC CTG GCT ACG GTC TCA AG
*ACSL4*	F: CAA TAG AGC AGA GTA CCC TGA G
	R: TAG AAC CAC TGG TGT ACA TGA C
*NCF2*	F: GAA GAT ACC TCT CCA GAA TCC G
	R: TTC TTA GAC ACC ATG TTC CGA A
*GPX2*	F: GTC ACT CTG AGG AAC AAC TAC C
	R: TTC TGA CAG TTC TCC TGA TGT C
*CBS*	F: GAA GCC TGG AGA CAC TAT CAT T
	R: CAT CAC GAT AAT GCA GCG ATA G
*LPCAT3*	F: CAT GAA AGT GTG GCT CTT TGA A
	R: GTT TGA AGA TGT AAC GGG CTA C
*GAPDH*	F: GGT TGT CTC CTG CGA CTTCA
	R: TGG TCC AGG GTT TCT TAC TCC

## Data Availability

The datasets and Supplementary Materials supporting this investigation are available within the published article and associated resources. Additional requests for data access may be submitted to the lead investigators. Transcriptomic profiles were sourced from the GEO repository (https://www.ncbi.nlm.nih.gov/geo/; accessed 1 October 2024), incorporating four datasets (GSE65114, GSE87473, GSE102133, and GSE186582) and single-cell RNA-seq data (GSE217695). Computational scripts generated during this research are available upon formal request to the corresponding authors.

## Supplementary Materials

The following supporting information can be downloaded at https://www.mdpi.com/article/10.3390/genes16050496/s1: Figure S1: Functional and pathway enrichment analyses were conducted. Biological processes linked to signature genes in the healthy cohort (A) and the IBD cohort (B) were delineated via GSEA. Molecular signaling cascades associated with signature genes in the healthy cohort (C) and the IBD cohort (D) were elucidated through GSEA; Figure S2: GSVA analysis of DRFGs GSVA analysis in IBD group of DUOX2. (A), ACSL4 (B), NCF2 (C), SOCS1 (D), GPX2 (E), ATF3 (F), CBS (G), LPCAT3 (H), and HSD17B11 (I); Figure S3: Batch Effect Analysis and Cell Type Identification in GSE217695 Dataset. (A) Batch effect analysis of the 18 samples in the GSE217695 dataset. (B) Identification of 11 distinct cell types based on clustering results and the expression of canonical surface markers. Table S1: Ferroptosis-associated genes (FAGs) sourced from the FerrDB repository; Table S2: Disulfidptosis-associated genes (DAGs) curated from Gan et al.’s dataset; Table S3: Marker Gene-Based Cell Type Annotation; Table S4: Significant Molecular Associations with Strong Correlations Identified in 371 Candidates; Table S5: Cross-Platform Consistency Validates Core Regulatory Roles in Colitis.
