# Supplementary material for: Development and Experimental Validation of Machine Learning-Based Disulfidptosis-Related Ferroptosis Biomarkers in Inflammatory Bowel Disease
Source: Genes (Basel). 2025 Apr 27;16(5):496. doi: 10.3390/genes16050496 (PMC12110833; doi:10.3390/genes16050496)
Supplement: Supplementary file 1 [file genes-16-00496-s001.zip › Figure S1.pdf]

Figure S1

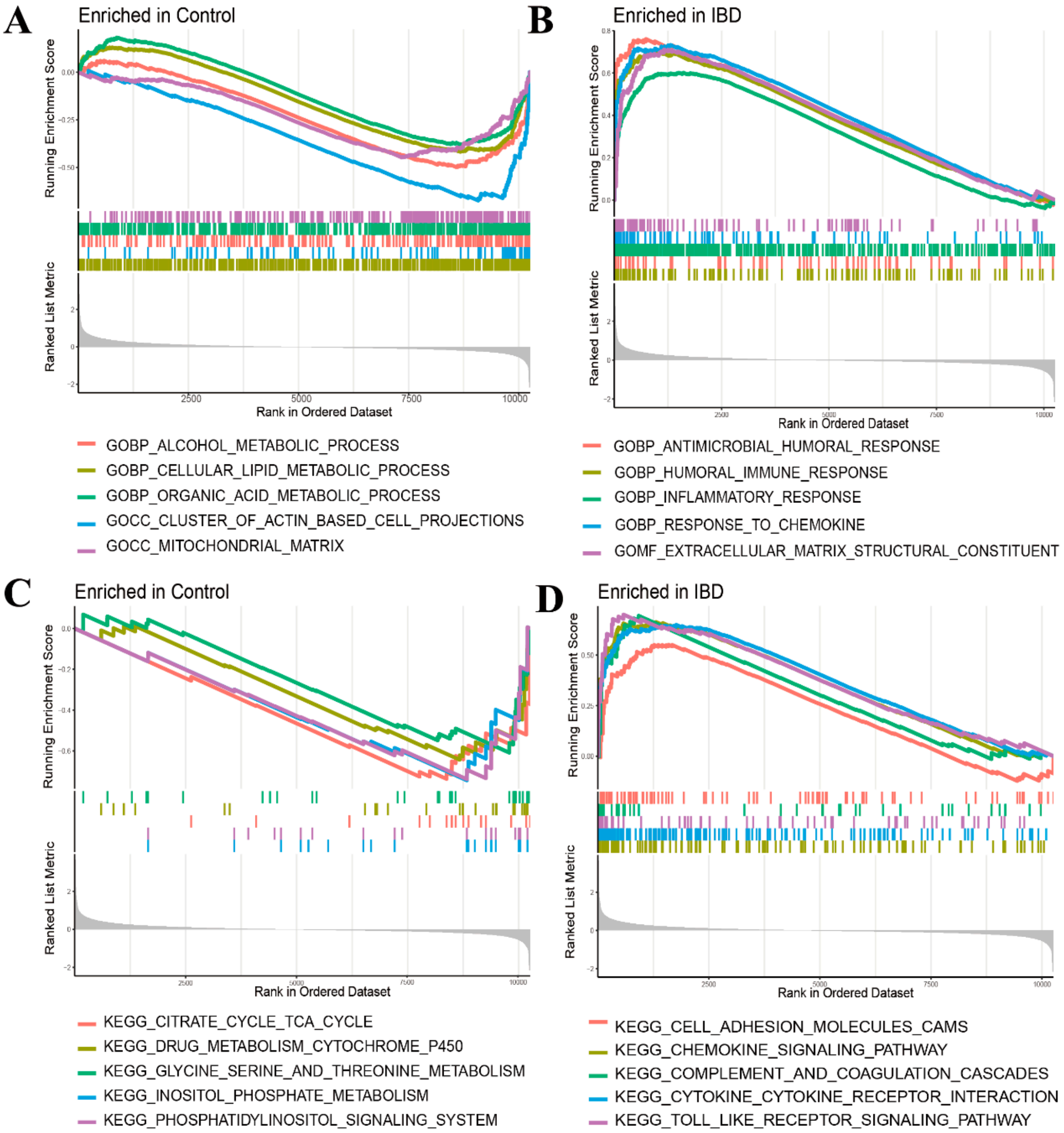

Figure S1. Functional and pathway enrichment analyses were conducted. Biological processes linked to signature genes in the healthy cohort (A) and the IBD cohort (B) were delineated via GSEA. Molecular signaling cascades associated with signature genes in the healthy cohort (C) and the IBD cohort (D) were elucidated through GSEA.
