# Supplementary material for: Development and Experimental Validation of Machine Learning-Based Disulfidptosis-Related Ferroptosis Biomarkers in Inflammatory Bowel Disease
Source: Genes (Basel). 2025 Apr 27;16(5):496. doi: 10.3390/genes16050496 (PMC12110833; doi:10.3390/genes16050496)
Supplement: Supplementary file 1 [file genes-16-00496-s001.zip › Figure S2.pdf]

Figure S2

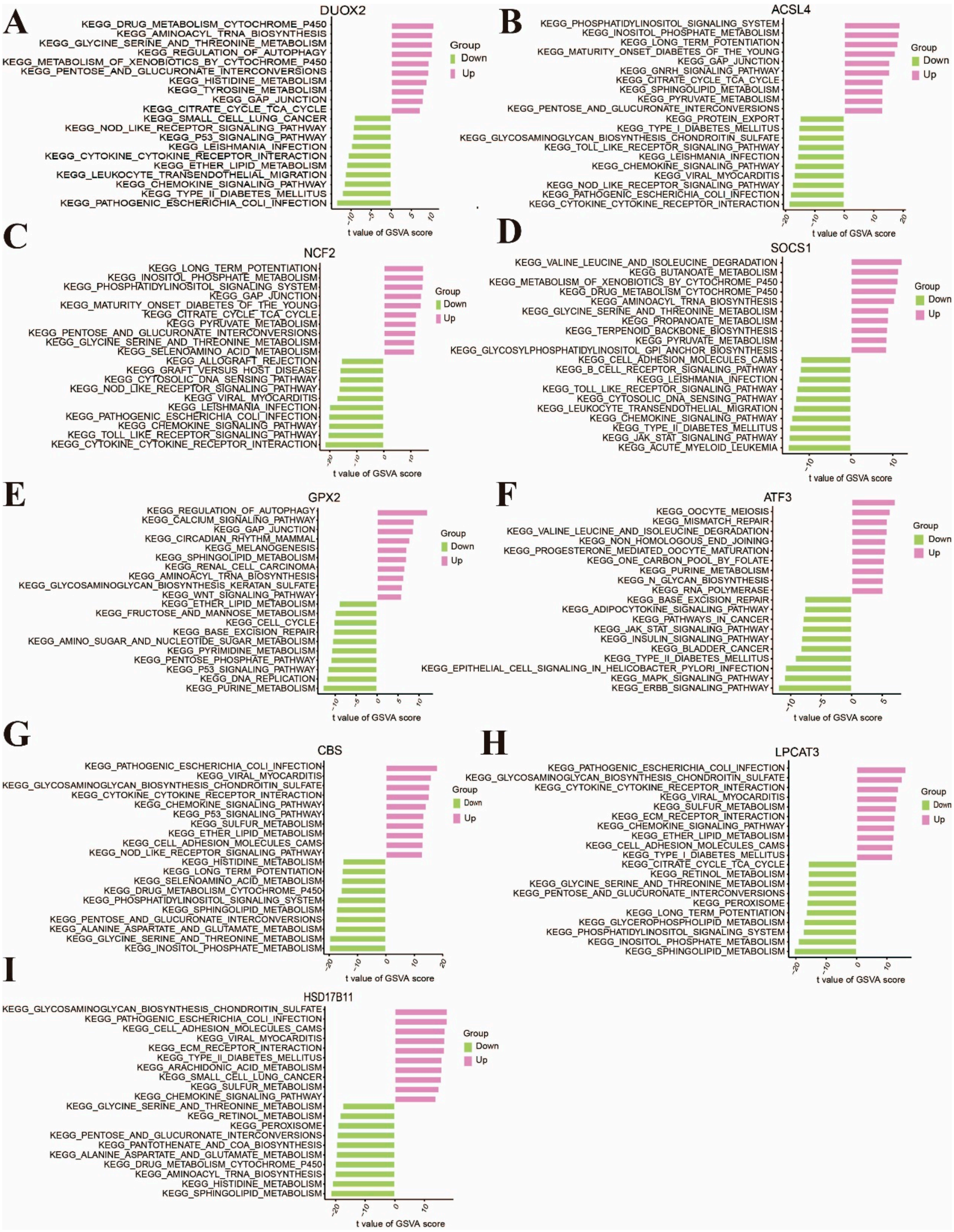

FigureS2. GSVA analysis of DRFGs GSVA analysis in IBD group of DUOX2. (A), ACSL4(B), NCF2(C), SOCS1(D), GPX2(E), ATF3(F), CBS(G), LPCAT3(H), and HSD17B11(I).
