# Supplementary figures and images for: Development and Experimental Validation of Machine Learning-Based Disulfidptosis-Related Ferroptosis Biomarkers in Inflammatory Bowel Disease
Source: Genes (Basel). 2025 Apr 27;16(5):496. doi: 10.3390/genes16050496 (PMC12110833; doi:10.3390/genes16050496)

**Figure S3**

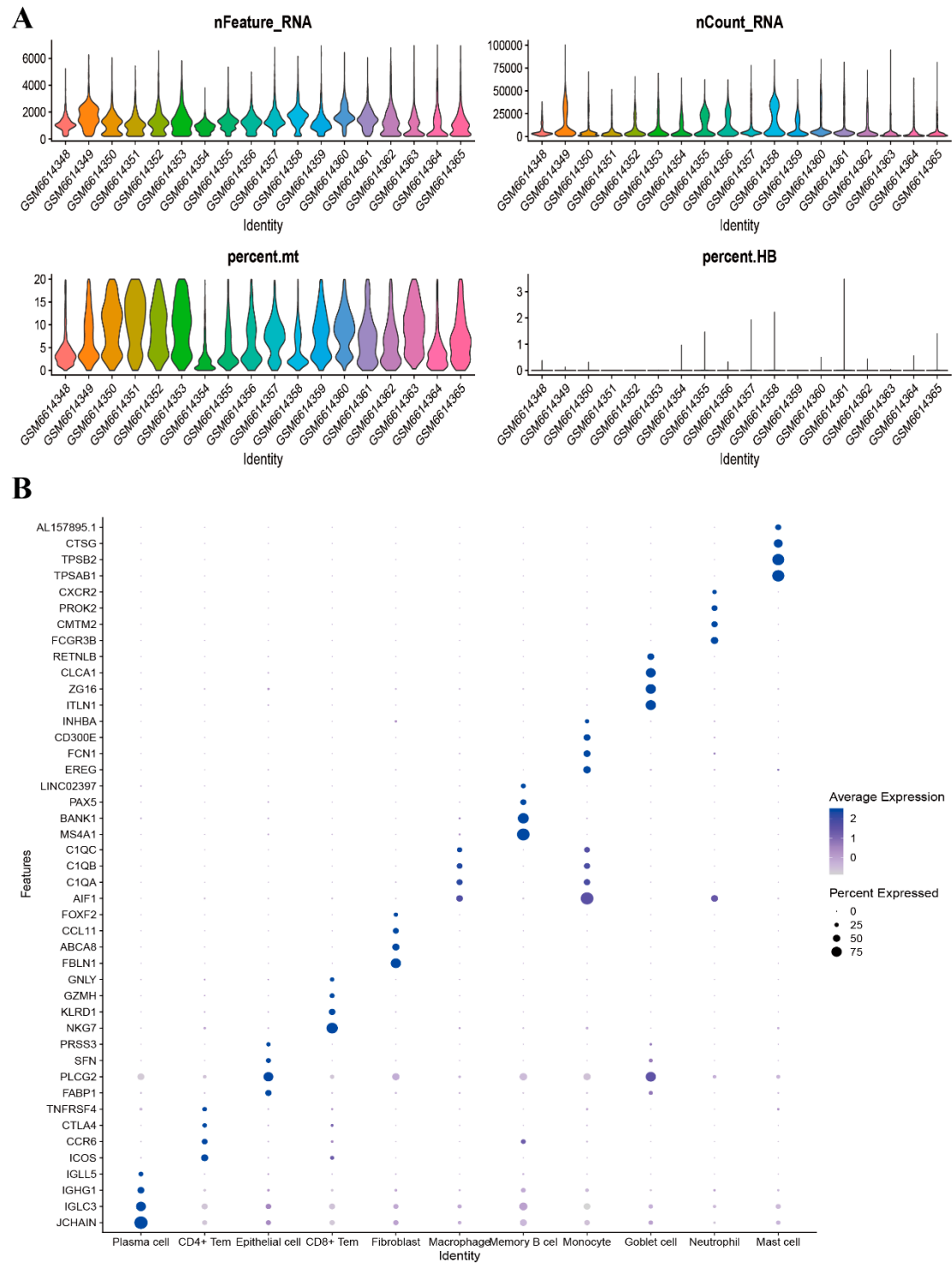

Supplement: Supplementary file 1 [file genes-16-00496-s001.zip › Figure S3.pdf]
